# Redefining Aging in HIV Infection Using Phenotypes

**DOI:** 10.1007/s11904-017-0364-x

**Published:** 2017-09-20

**Authors:** David M. Stoff, Karl Goodkin, Dilip Jeste, Maria Marquine

**Affiliations:** 1 AIDS Research Training-Health Disparities and HIV Aging/Comorbidity Research Programs, Division of AIDS Research, National Institute of Mental Health, 5601 Fishers Lane Room 9E25, MSC 9831, Bethesda, MD 20892 USA; 2 East Tennessee State University, Johnson City, TN USA; 3 University of California San Diego, La Jolla, CA USA

**Keywords:** HIV, Phenotypes, Aging, Comorbid aging, Cognitive aging, Interventions

## Abstract

**Purpose of review:**

This article critically reviews the utility of “phenotypes” as behavioral descriptors in aging/HIV research that inform biological underpinnings and treatment development. We adopt a phenotypic redefinition of aging conceptualized within a broader context of HIV infection and of aging. Phenotypes are defined as *dimensions* of behavior, closely related to fundamental mechanisms, and, thus, may be more informative than chronological age. Primary emphasis in this review is given to comorbid aging and cognitive aging, though other phenotypes (i.e., disability, frailty, accelerated aging, successful aging) are also discussed in relation to comorbid aging and cognitive aging.

**Recent findings:**

The main findings that emerged from this review are as follows: (1) the phenotypes, comorbid aging and cognitive aging, are distinct from each other, yet overlapping; (2) associative relationships are the rule in HIV for comorbid and cognitive aging phenotypes; and (3) HIV behavioral interventions for both comorbid aging and cognitive aging have been limited.

**Summary:**

Three paths for research progress are identified for phenotype-defined aging/HIV research (i.e., clinical and behavioral specification, biological mechanisms, intervention targets), and some important research questions are suggested within each of these research paths.

## Introduction: Aging as a Multidetermined Process-The Phenotype Concept

During this era of unprecedented antiretroviral therapeutic efficacy, there is hope for successfully treating people aging with HIV infection, but this has been compromised by the changing clinical pattern of HIV infection with the emerging aging comorbidities and other manifestations (e.g., accelerated aging). This has provided new challenges relating to the care and management of aging patients. In the aging person, these new challenges are frequently expressed as “phenotypes” and are most often viewed within the now well-accepted notion of HIV infection as a chronic disease. Before we can reach deeper mechanistic insights or develop more effective interventions in aging/HIV-infected individuals, it is essential to rethink how we define aging in this context,

In aging research, use of the categorical variable chronological age *by itself* may provide limited, and sometimes misleading, information due to variability of the aging process both within and between populations. Aging is a heterogeneous process and spans various domains including inflammatory aging, immunosenescence, metabolic aging, cognitive aging, and psychosocial aging. Research studies on aging/HIV should go beyond using chronological age alone because the process of HIV infection, like the process of aging, is shaped by the individual’s social, physical, cultural, and economic setting in addition to biological senescence. In this context, aging is a developmental biological concept within an integrative lifespan framework, which promotes understanding of how and why individuals change [1, 2].

Other than aging as a biological process separate from chronological age, it has been noted [3] that the chronological age variable in HIV research has hindered understandings of factors that influence the course and treatment of HIV among older adults. Indeed, the categorical aging approach ignores the view that aging is continuously distributed through the general population via “phenotypes.” This paper adopts a phenotypic framework wherein aging is conceptualized within a broader context of objectively determined phenotypic manifestations (e.g., comorbidities) with the passage of time.

The phenotype construct offers multiple advantages in HIV/aging research. “Phenotypes” are *dimensions* of behavior, closely related to fundamental mechanisms, and, thus, may be more informative than chronological age, especially in HIV research. Phenotypes have been used to identify the vulnerable subsets of the aging population—frailty [4], comorbidity [5, 6], and disability [7]. Future developments depend on identifying narrower phenotypes with greater clinical validity as treatment targets.

This article critically reviews the phenotype construct within the context of aging and HIV infection. Phenotypes have been studied far more often in gerontology [8] than in HIV, and the integration of these two areas needs to be improved. For example, frailty has been studied rather extensively in gerontology [9]; yet, the application to HIV has been limited. We highlight behavioral research of phenotypes in aging, focusing primarily on comorbid and cognitive aging because of relevance to mental health and published literature on these phenotypes. We cover the following: definition and characteristics of comorbid aging and cognitive aging phenotypes (Comorbid Aging/HIV and Cognitive Aging/HIV: Defining and Characterizing); other phenotypes (frailty, disability, accelerated aging, successful aging) (Other Aging Phenotypes in HIV Infection); relationships among overlapping phenotypes (Phenotypic Inter-Relationships: Comorbid Aging/HIV and Cognitive Aging/HIV); current evidence for comorbid aging and cognitive aging targeted interventions (Interventions Targeting Aging/HIV-Related Phenotypes); and summary/research directions (Summary and Directions).

## Comorbid Aging/HIV and Cognitive Aging/HIV: Defining and Characterizing

“Phenotypes” are used as behavioral descriptors of aging/HIV that may have different disease courses, biological underpinnings, and treatment responses. The major aging/HIV-related phenotypes are defined in Table 1.

**Table 1:** Definitions of HIV-relevant phenotypes in older persons

Phenotype	Definition	Illustrative HIV study
Disability	Dependency or difficulty in carrying out activities essential to independent living	Avila-Funes et al. 2016 [10]
Frailty	Physiologic state of increased vulnerability to stressors	Gustafson et al. 2016 [11]
Comorbid aging	Concurrent presence of two or more medically diagnosed diseases in the same person	Rodriguez-Penney et al. 2013 [12]
Cognitive aging	Continuum of cognitive decline across domains of information processing speed, episodic and working memory, attention, abstraction/executive function, motor function, visuospatial function, and language	Fazeli et al. 2014 [13]
Premature or accelerated aging	Conditions associated with aging process that occur at an earlier age or may involve high life stressor burden, lack of social support, and/or maladaptive coping strategies	Sheppard et al. 2017 [14]
Successful aging	Characterized by subjective well-being and satisfaction with life, associated with resilience and other positive traits.	Moore et al. 2013 [15]

Phenotypes are defined as subsets of biological factors that may define sub-groups of aging individuals (also relevant to HIV infection). We use “endophenotypes” to provide stratifications by sub-group (behavioral category or associated biomarker), to optimize intervention targeting and inform clinical trials addressing abnormalities in key biological systems. We did not cover phenotypes of biological aging, such as the immune risk phenotype, reflecting immunosenescence associated with markers of immune aging like the depletion of naïve CD4 cells [16] and the exhaustion of cytotoxic T cell proliferation. Endophenotypes may shed light on fundamental aging processes [17] and may be related to clinical progression profiles of HIV [18].

### Comorbid aging/HIV

Comorbidities in HIV infection, in the broadest sense, consist of a spectrum of conditions including medical (e.g., cardiovascular disease, diabetes, hypertension, osteoporosis, hepatic and renal disease, chronic respiratory disorders, cancers, HCV co-infection), neuropsychiatric comorbidities (e.g., substance use, depressive, and sleep disorders), and social comorbidities (e.g., educational disadvantage, poverty, chronic trauma, illiteracy) [19]. The extended exposure of older adults to both HIV and antiretroviral drugs appears to increase their risk of illness and death from HIV-associated non-AIDS (HANA) comorbidities, such as cardiovascular disease (including myocardial infarction [MI]), cerebrovascular accident [CVA], and other aforementioned illnesses not directly associated with HIV infection [20]. HANA comorbidities affect timely initiation of ART in older adults, suggesting the need for improved coordination and integration of care and management of prescription practices [21]. Previously described findings of racial disparities in the quality of care for persons with HIV infection [22] have been extended to common comorbidities suggesting the need for implementation of cross-cutting multilevel interventions [23].

The largest epidemiological study (Veterans Administration Cohort Study, VACS) addressing the prevalence of HANA comorbidities in HIV indicated a prevalence of 60–63% [20]. Some investigators have reported a yet higher prevalence of 94%, with an average of three comorbid conditions in HIV-infected persons over age 50 living in the USA [12]. Furthermore, among older HIV-infected individuals, increased psychiatric comorbidities are linked to decreased quality of life [24]. The neuropsychiatric aspects of HIV and aging have been reviewed elsewhere [25].

Major depressive disorder (MDD) is the most common psychiatric diagnosis in HIV/AIDS at a prevalence over twice the rates seen in the general population [26, 27]. However, whether older HIV-infected adults experience higher rates of MDD than younger adults is unclear. Some studies report depression rates increased with age [28], while others report absence of the usual age-dependent decline in depression for older HIV-infected adults [29]. Further, it has been suggested that MDD is an independent risk factor for heart failure in HIV+ adults reinforcing the importance of identifying and managing MDD among HIV+ patients and of identifying interventions to reduce cardiovascular morbidity and mortality in those with both HIV and MDD [30].

Substance use disorder comorbidities are also common in older patients and put them at higher risk for HIV infection. Substance use accounts for over 16% of new HIV infection in people aged 50 and older [31]. Unlike the general population where substance use disorder rates decline in people age over 50, older HIV-infected patients maintain steady rates of substance use disorder [28, 29]. In more detailed analyses, patterns of recent substance use were associated with varying levels of HIV medication adherence and perceived substance use impairment, indicating that substance type matters when considering the health of aging adults living with HIV and that multiple-substance use needs to be addressed by interventions aimed at improving medication adherence [32]. Current and lifetime alcohol and substance use disorders may be compounding brain dysfunction, specifically in older HIV-infected individuals. Thus, a study [33] found that there was a legacy effect in older methamphetamine-using HIV seropositive individuals compared to their younger counterparts. Another study [34] found that lifetime alcohol use disorder and old age were associated with significant reduction in brain volumes.

### Cognitive Aging/HIV

Approximately 50% of persons living with HIV suffer from impaired cognition, a frequency that increases with age [35]. Moreover, impaired cognition has adverse impact on everyday functioning and quality of life [36]. Clinically, HIV-associated neurocognitive impairment (NCI) is associated with a variety of symptoms, including problems in attention, concentration, learning, memory, psychomotor ability, and speed of information processing. There is considerable heterogeneity in the cognitive patterns among HIV-infected individuals, requiring a focus on distinguishing components of the cognitive aging phenotype. HIV infection often results in varying degrees of neurocognitive dysfunction, ranging from asymptomatic NCI to HIV-associated dementia (HAD), collectively termed HIV-associated neurocognitive disorders [37]. The nature of HIV-associated NCI has evolved considerably over time, particularly the spectrum of neurocognitive disorders in the era of combination antiretroviral therapy (cART) [38]. As widely reported elsewhere, there has been a significant decline in HAD but a higher persistence of the milder categories, with frequently cited percentages of 2% for HAD and as much as 50% for mild neurocognitive disorder taken together with asymptomatic neurocognitive disorder [37, 39]. Thus, further endophenotypic separation within the cognitive phenotype in HIV clinical conditions has assumed particular importance. Additionally, there has been a shift pertaining to types of cognitive domains that appear to be most notably impacted [38]. In the pre-cART era, HIV-infected individuals were more likely to demonstrate impairment in motor skills, information processing speed, and verbal fluency, whereas patients receiving cART today may be more likely to exhibit difficulties with memory and executive functioning [38]. Also relevant is the notion that there might be different trajectories of cognitive change over time among HIV seropositive persons. In a recent multi-site study of longitudinal neurocognitive change in HIV-infected persons living in the USA, most remained stable (61%), nearly a quarter declined (23%), and others improved (17%) cognitively [40]. Neurocognitive change appears to be driven by a complex set of risk factors involving HIV disease, its treatment, and comorbid conditions, and favorable neurocognitive outcomes may be achieved by instituting cART early [40]. Consistent with these findings, others have shown that cognitive declines may not necessarily occur in all HIV seropositive persons, suggesting that future interventions need to target distinct cognitive sub-groups. More specifically, two clusters have been described with a lower performing cluster exhibiting poorer performance across all domains (except psychomotor speed) and a “normal” cluster displaying similar performance as the HIV seronegative group [13]. Using longitudinal data from the MACS cohort, there were deleterious effects of aging and more severe stage of HIV infection on neurocognitive function, generally, with an additional deleterious interaction effect on episodic memory and motor performance [41].

## Other Aging Phenotypes in HIV Infection

We briefly discuss other aging phenotypes that have not been studied in the same depth in HIV infection as comorbid and cognitive aging, the focus of our review. Accepted definitions for all HIV-related aging phenotypes are presented in Table 1.

Both primary types of *disability* (i.e., difficulty in basic and instrumental activities of daily living) among older HIV-infected patients are independently associated with older age and HIV disease burden (i.e., lower CD4 cell count and detectable viral load) [42]. Over the last two decades, multiple objective tools to assess functional impairment among people with HIV have made important contributions to our understanding of functional limitations and the disability process in HIV/AIDS settings [42]. Disability is thought to result from multi-system dysfunction such that an underlying pathology may lead to impairment at the tissue, organ, or body system level which results in a “functional limitation” and ultimately leads to disability [42].

Among all the aging phenotypes, the *frailty* phenotype is considered the most well-operationalized and characterized [9, 43]. Recognition of the similarities in the biology and clinical manifestations between older adults and HIV-infected adults led to the study of frailty in HIV infection [42, 44]. Multiple studies demonstrate increased frailty with age in HIV infection, particularly among persons with increased chronic comorbid disease or with cognitive decline (discussed in next section on interrelationships). Although many studies have tested clinical interventions on components of the frailty phenotype (i.e., strength, activity), few studies have tested clinical interventions to reduce frailty as an aggregate syndrome in any population or group [44]. In HIV infection, there have been few frailty-targeted interventions, and future frailty intervention research will depend on better understanding frailty biology as well as untangling the relationships of frailty, disability, comorbidity, and cognition.

Research on the *accelerated aging* phenotype suggests that a more rapid aging process is taking place in HIV infection, and there is debate as to whether the outcomes examined represent an acceleration of the typical aging process or a greater accentuated risk for age-related declines [45]. Intervention studies must reflect the data that many age-related phenotypes present earlier and more aggressively and progress more rapidly in HIV-infected patients compared with the general population. Regardless of the mechanism, this phenotype occupies a central role interacting with multiple HIV-related aging phenotypes since multiple studies have shown that in HIV infection, accelerated aging interacts with cognitive aging, comorbid aging, and frailty (discussed in next section on interrelationships). Future HIV/accelerated aging research should use both multivariate methods and longitudinal design to determine whether HIV-infected individuals who evidence an accelerated aging profile in their 50s and 60s are at increased risk for more severe impairments as they age into their later 60s and 70s.

Although aging with HIV infection is often reportedly accompanied by increases in patience, contentment, moderation, wisdom, and a greater respect for health and life [46] as well as resilience and increased personal strength [47], research on the *successful aging* phenotype in people with HIV has been scarce. Few HIV-infected persons, let alone those with AIDS, would meet the definition of successful aging proposed by Rowe and Kahn [48], i.e., absence of objective physical, cognitive, and psychosocial disability, partly because of being infected by HIV. However, that definition has been challenged and replaced with a notion of successful aging as subjective well-being together with the findings that resilience, optimism, and sense of personal mastery are more closely associated with self-rated successful aging than duration or severity of HIV infection [15]. Thus, “successful aging” may coexist with other aging phenotypes and possibly influence them. Indeed, successful aging is not uncommon in people living with HIV wherein resilience is a predictor of emotional well-being and psychological adjustment [49].

## Phenotypic Inter-Relationships: Comorbid Aging/HIV and Cognitive Aging/HIV

While aging-related phenotypes (i.e., comorbidity, frailty, disability) may represent distinct clinical entities and have their own unique challenges, there are considerable inter-relationships among phenotypes [50]. Considering interacting functions and the heterogeneity of underlying mechanisms, especially in the context of the multi-determined nature of HIV infection and of the aging process, is of critical significance to generate a comprehensive profile of HIV-related phenotypes in aging individuals. Phenotypic overlap in aging is also partially consistent with a related view of overlapping patterns of multi-morbidity (i.e., two or more co-occurring chronic conditions not linked to the index condition), wherein patterns or clusters of chronic conditions have been described in the elderly [51]. Additionally, the existence of overlapping phenotypes serves as a strong rationale for the development of integrated interventions. Table 2 presents studies on inter-relationships between comorbid and cognitive phenotypes in older HIV-infected adults. Phenotypic inter-relationships were of two main kinds: (1) correlational (i.e., associative) and (2) temporal (i.e., predictability, vulnerability).

**Table 2:** Aging-related phenotype interrelationships in HIV-infected older adults

Comorbid aging with cognitive aging: Correlative Relationships	Older HIV-infected Sample	Study
Neuropsychiatric symptoms (psychiatric symptom burden using Geriatric Depression Scale and Neuropsychiatric Inventory Questionnaire) not associated with cognitive functioning (HAND, Clinical Dementia Rating)	Older (> 60 years) HIV infected adults from community	Milanini et al. 2017 [52]
Increasing number of medical comorbidities (most common hyperlipidemia, hypertension, peripheral neuropathy) associated with increasing risk of having more geriatric syndromes (among cognitive and functional impairment, depression, falls, frailty, sensory impairment)	Older (> 50 years) HIV negative infected adults	Greene et al. 2015 [53]
Comorbid medical disorders (i.e., hypertension, stroke) associated with lower performing cognitive subtype (processing speed), more prominent in older adults, but not with normal performing cognitive subtype	HIV-infected adults and older adults (mean age 46.1 yrs)	Fazeli et al. 2014 [13]
Comorbidities (diabetes most prevalent) associated with global and syndromic neuropsychological impairment (interfering with daily functioning) in older group	Older (≥ 50 years) and younger (≤ 40 years) HIV-infected and uninfected from community	Rodriguez-Penney 2013 [12]
Depressive symptoms (Beck Depression Inventory) associated with poorer selective cognitive (motor speed visuospatial memory) and life functioning (quality of life)	Middle aged men and women HIV infected (43.9 years mean age)	Sassoon et al. 2012 [54]
Depressive symptoms (Beck Depression Inventory) associated with neuropsychological impairment in younger but not in older adults	HIV infected older adults (< 50 years) from Hawaii Aging with HIV Cohort and HIV infected younger adults (20–39 years)	Shimizu et al. 2011 [55]
Cardiovascular comorbidities (carotid disease) associated with some measures of cognitive impairment (Stroop interference)	Middle aged women (early 40s) from WIHS	Crystal et al. 2011 [56]
Increasing degree insulin resistance associated with lower cognitive performance (neuropsychological summary scores), especially in middle-aged and older subgroup	Middle-aged and older (> 50 years) participants from Hawaii Aging HIV Cohort	Valcour et al. 2006 [57]
Increased depressive symptoms associated with increased self-reported memory problems	HIV positive veterans in care (< 50 years) from Veterans Aging Cohort Five-Site Study	Justice et al. 2004 [28]
Comorbid aging with cognitive aging: Temporal Relationships	Older HIV-infected sample	Study
Comorbidity risk factors (stimulant use, vascular disease, hepatitis C, HIV disease severity, cognitive reserve) predict cognitive functioning (learning/memory, verbal fluency); cognitive reserve strongest predictor of neurocognition; impact on learning/memory and verbal fluency in older and in younger adults linked to working memory and executive function	HIV infected older adults (> 50 years) on HAART, recruited from community	Patel et al. 2013 [58]
Depression was strongest predictor of cognitive functioning; those with higher levels of depressive symptomatology experienced poorer cognitive functioning.	HIV-infected, community-dwelling older adults (mean age 75 years)	Vance et al. 2005 [59]
Comorbid aging with frailty relationships: Correlative Relationships	Older HIV-infected sample	Study
Aging related chronic diseases and comorbidities (e.g., hypertension, diabetes, cancer) associated with HIV infection related sociodemographic factors and health behaviors	Middle-aged women living with HIV from the WIHS cohort	Gustafson et al. 2016 [11]
Multiple chronic medical conditions, depression symptoms, and inflammatory index associated with frailty (Fried phenotype criteria); inflammatory index independently associated with frailty and mortality risk among older infected and noninfected injection drug users	Hopkins ALIVE cohort (Median age 47 yrs., 29% HIV infected)	Pigott et al. 2015 [60]
Medical comorbidities/depression and adverse health outcomes (hospitilization/mortality) associated with frailty and with risk for hospitilization	HIV infected and uninfected from Veteran Administration Cohort Study, VACS (40–60 years)	Akgun et al. 2014 [61]
Hepatitis C seropositivity, history of AIDS-defining-illness, and greater depressive symptoms more likely to be associated with pre-frail/frail participants	Observational cohort of HIV-infected persons (median age 47 yrs), 95% on cART	Onen et al. 2014 [62]
Most common comorbidities were hypertension (60%) and those with a history of AIDS-related opportunistic infections; comorbidities, cognitive impairment more prevalent in severely frail	Frail older (> 60 years) patients in three groups (mildly, moderately, severely frail)	Ruiz and Cefalu 2011 [63]
Comorbid aging with frailty Relationships: Correlative Relationships	Older HIV-infected sample	Study
Medical comorbidities predict frailty with the strongest predictability for hepatitis C infection, depressive symptoms, history of diabetes, and kidney disease (in decreasing order of predictability); sizable proportion of HIV+ and HIV- men had positive frailty status suggesting comorbidities and frailty are not synonymous	HIV-infected MSM (men who have sex with men) from MACS (Multicenter AIDS Cohort Study) (median age 53.8 yrs); 84% on HAART)	Althoff et al. 2014 [64]
Comorbid aging with disability: Correlative Relationships	Older HIV-infected sample	Study
Increased number of chronic diseases (dyslipidemia 57%, and hypertension 33%, most frequent), age, VL and CD4 count were independently associated with both types of disability (basic and instrumental activities of daily living)	HIV infected older adults (mean age, 59.3 years) receiving ambulatory care in HIV clinic Mexico	Avila-Funes et al. 2016 [10]
Depression and sleep problems strongly associated with WHO disability assessment scores (domains of cognition, mobility, self care, getting along, life activities, participation)	HIV-infected older adults (mean age, 65 years) from Uganda	Mugisha et al. 2016 [65]
Pain independently associated with substantially increased odds of impairment in three domains of physical function (mobility, self care, usual activities)	HIV-infected middle aged adults (median age, 43.6 years); outpatients	Merlin et al. 2013 [66]
Medical comorbidities (Charlson) independently associated with physical (and mental) health related quality of life	Older (≥ 50 years) and younger (≤ 40 years) HIV-infected and uninfected from community	Rodriguez-Penney et al. 2013 [12]
Major depression, chronic lung disease, coronary artery disease, hypertension, smoking independently associated with reduced physical function (i.e., comorbidities account for relationship between increased age and reduced physical function in both infected and uninfected)	HIV-infected adults from VACS (< 40, 40–69, 60–59, and > 60 years)	Oursler et al. 2006 [67]
Comorbid aging with accelerated aging relationships: Correlative Relationships	Older HIV-infected sample	Study
Prevalence of two or more non-infectious comorbidities (among diabetes mellitus, cardiovascular disease, bone fractures, and renal failure) similar in HIV-infected patients aged 41–50 years and uninfected persons 51–60 years	HIV-infected and uninfected adults from Italy (< 40, 41–50, 51–60, and > 60 years)	Guaraldi et al. 2011 [68]
Non-AIDS defining cancers (anal, colon) diagnosed in significantly younger persons (by about 20 yrs) with AIDS than expected in the general population	Persons with AIDS in the U.S. population-based HIV/AIDS Cancer Match Registry (restricted to age-at-diagnosis distributions 4–60 months after AIDS diagnosis)	Shiels et al. 2010 [69]
Cognitive aging with frailty relationships: Correlative Relationships	Older HIV-infected sample	Study
Cognitive impairment (47%), difficulty with instrumental activities of daily living (46%) and prefrailty (56%) most frequent geriatric syndromes; also experienced at younger ages	HIV-infected adults on HAART (median age, 57 years)	Greene et al. 2015 [53]
Trend toward lower memory functioning (but not executive function, speed of processing, motor skills), together with decreased grip strength in older adults (> 50 years); comorbid depression did not differ between older and younger groups	Small groups of older (mean age, 56.5 years) and younger (mean age, 31.5 years) HIV-infected adults)	Sandkovsky et al. 2013 [70]
Many cognitive domains (such as verbal and visual memory, visual perception, and language) significantly related to aerobic fitness; More severe forms of HIV-associated neurocognitive disorders (HAND) such as mild neurocognitive disorder and HIV-associated dementia associated with reduced likelihood of higher aerobic fitness	HIV-infected adults (mean age, 58.9 years) on HAART from infectious disease clinic	Mapstone et al. 2013 [71]
Cognitive aging with disability: Correlative Relationships	Older HIV-infected sample	Study
Reduced speed of processing associated with poorer everyday functioning in HIV infected older adults; chronicity of HIV infection had stronger relationship with poorer speed of processing than with poorer everyday functioning	Older (mean age, 57 years) and younger (mean age, 42 years) HIV infected and uninfected adults	Vance et al. (2013) [72]
Most cognitive measures (e.g., trail making, finger tapping, letter and pattern comparison, digit span) associated with poorer performance on instrumental activities of daily living	Older (mean age, 45 years) and younger (mean age, 38 years) infected and uninfected adults recruited from community	Vance et al. (2011) [73]
Cognitive aging with accelerated aging: Correlative Relationships	Older HIV-infected sample	Study
Auditory attention of older HIV+ adults impaired relative to age matched seronegative adults (accentuated aging) and impaired to same degree as age matched seronegative adults in their 70’s (accelerated aging); no association with memory, language, or speeded executive functions.	Older HIV-infected and uninfected adults (aged > 65 years and from 50 to 65 years)	Sheppard et al. 2017 [14]
HIV-associated neurocognitive disorder, HAND, diagnoses associated with accelerated epigenetic aging (average relative acceleration of 3.5 years) in post-mortem brain tissue (occipital cortex); age acceleration not correlated with pre-mortem neurocognitive functioning or HAND severity	Patients with HAND and neurologically normal (mean age, 48.5 years at death)	Levine et al. 2016 [74]
HIV-associated neurocognitive disorder, HAND, and neurocognitive impairment scores (global and domains motor, memory encoding and retrieval, executive function) with alterations in CSF metabolites (glutamate, NAA), plasma inflammatory biomarkers associated with advanced age	CSF analyses of older (> 50 years) and younger (< 50 years) infected and uninfected adults from National NeuroAIDS Tissue Consortium, NNTC	Cassol et al. 2014 [75]
Age-related metabolic changes affecting cardiovascular risk hypertension) predispose to neurocognitive decline (psychomotor speed) through accelerated cerebrovascular disease	Middle-aged (40 years) adults HIV infected and uninfected, from Multicenter AIDS Cohort Study, MACS, cardiovascular and cerebrovascular disease substudy	Becker et al. 2009 [76]
Cognitive aging with successful aging: Correlative Relationships	Older HIV-infected sample	Study
One third of older persons with HIV free of cognitive impairments on battery of validated cognitive tests (also had better everyday functioning and fewer medical and psychiatric comorbidities)	Middle-aged and older persons (mean age 51 years) infected with HIV	Malaspina et al. 2011 [77]

### Relationships Between Comorbid Aging/HIV and Cognitive Aging/HIV

Medical comorbidities occur earlier and more frequently in older persons living with HIV infection; some of its consequences include neurocognitive and functional impairment, frailty, organ system failure, and increased hospitalizations [78]. The highly prevalent medical comorbidities further complicate assessment, diagnosis, and interventions for HIV-associated neurocognitive disorder (19). More specifically, a greater medical comorbidity burden (mostly for diabetes and malignancy), assessed by the Charlson Comorbidity Index [79], in older HIV-infected adults was associated with global NCI and related functional declines and lower physical health-related quality of life [12]. Other data in older HIV-infected adults are supportive of this finding—reporting that these individuals develop metabolic dysfunction (and other comorbidities) disorders leading to type 2 diabetes and NCI, through depleting cognitive reserve, that can impair everyday functioning and reduce quality of life [80]. In line with these findings, changes in VACS Index scores, which is a composite marker of HIV disease severity, including age, HIV-related factors (current plasma HIV RNA load and CD4 cell counts) and “non-HIV-associated biomarkers” (indicators of renal and hepatic function, anemia, and HCV co-infection), correspond to longitudinal changes in neurocognitive function among middle/aged HIV-infected individuals [81].

Most of the empirical overlapping relationships between comorbid and cognitive aging are associative, with few studies demonstrating temporal relationships (Table 2). These correlational relationships may potentially be attributed to factors that are causal (i.e., one phenotype increases risk for the other), genetic (i.e., heritable genetic factors or common liability for familial aggregation), or phenomenological (i.e., shared behavioral features). Direct causality cannot be inferred from these correlational studies, as effects may be due to interactions of independent components. One hypothesis to explain the comorbidity/cognition interactions is that comorbidities pose an additional burden on the cognitive functioning of HIV-infected individuals so that cognitive deficits are exacerbated by the presence of comorbidities. This interaction may be evident in aging/HIV-infected individuals because of the co-occurrence of aging-related conditions. These comorbidities and associated cognitive impairment may also be attributable to the chronic HIV-induced inflammatory state [82]. HIV-associated comorbidities and inflammation may accelerate the aging process, which may detrimentally impact everyday functioning via declines in cognitive functioning [83]—highlighting the importance of the “accelerated aging” phenotype. In this view, comorbid and accelerated aging phenotypes may have causal consequences on other aging phenotypes. Other possibilities for the adverse impact of HIV-associated mental health comorbidities on cognition include (1) competing for cognitive resources through rumination (i.e., depressive or suicidal thoughts that prevent other thoughts from emerging), (2) displaying different cognitive profiles (e.g., executive dysfunction is common in schizophrenia), and (3) a convergence of compromising vascular effects (diabetes mellitus, hypertension, and HIV infection). Related to these possibilities, models have been proposed for the synergistic effects of HIV, type 2 diabetes, and aging on cognition [80]. To further elucidate the role of each of these mechanisms would require separating out components and domains of the cognitive phenotype.

### Relationships Between Comorbid Aging/HIV or Cognitive Aging/HIV with Other Phenotypes/HIV

Comorbid aging or cognitive aging also overlap with other phenotypes (i.e., frailty, disability, and accelerated aging), but they are distinctive in several respects. Correlated associative phenotypic findings are widespread, and few studies have explored their temporal likelihood of developing or how they arise and evolve (Table 2). The only exceptions to the rule of associative relationships are comorbidity predicting frailty [64] and cardiovascular comorbidities leading to cognitive decline [76]. Among a large cohort of men who have sex with men (MSM) from the Multicenter AIDS Cohort Study (MACS), medical comorbidities predicted the frailty phenotype with the strongest predictability for HCV co-infection, depressive symptoms, history of diabetes, and renal disease (in decreasing order of predictability) [64], leading to the conclusion that comorbidities and frailty are not synonymous; rather, comorbidities are potential etiologic agents for frailty, as originally hypothesized [9]. Thus, these phenotypes are most likely not orthogonal to one another but rather they will overlap in certain respects requiring further research to dissect them out and maintain construct validity across various older HIV-infected samples. Aging-related phenotypes may also be used to generate a multi-factorial vector toward deleterious aging outcomes of interest and are best studied in this combined fashion.

An issue of concern for HIV phenotype research is that these aging phenotypes have been identified, by and large, in non-HIV-infected populations, so that they must now be reassessed and evaluated in vulnerable HIV-infected populations (e.g., MSMs). This limits generalizability to other aging HIV-infected populations. To date, most studies examining comorbid and/or cognitive aging in the HIV-infected population have been cross-sectional in nature. The temporal relationship between aging-related phenotypes is not clearly understood, and longitudinal data are needed to better describe the trajectory of cognitive decline and how specific comorbidities contribute to other aging-related phenotypes. Finally, it is unclear from the current results how long-term cART may affect gait speed decline. This is an important question, as nearly all of the aging/HIV-infected individuals studied were receiving cART, and the majority were virologically suppressed—characteristics that are likely to be similar in most populations aging with HIV infection.

## Interventions Targeting Aging/HIV-Related Phenotypes

Typically, HIV behavioral interventions for older adults have employed chronological age to identify this sub-population. To date, little attention has been paid to interventions targeting HIV-related aging phenotypes [84].

### Comorbid Aging/HIV

Until recently, the older HIV-infected population with comorbid conditions has been largely ignored in intervention research because patients with significant comorbid disease have frequently been excluded. Clinical practice guidelines are more often single-disease focused, usually based on results from clinical trials without significant comorbid disease [85]. Consequently, many questions remain [78] regarding the clinical care of older HIV-infected patients, tolerability of ART in patients with multiple comorbidities, pharmacodynamics and pharmacokinetics in older patients, and interactions between ART and co-administered drugs targeting comorbidities. HIV comorbidities might impact the disease process since these comorbidities may interfere with the receipt and timing of ART initiation, disrupt ART metabolism, or require drug treatment that may interact with ART, complicating HIV/AIDS disease treatment and survivability [86, 87]. While HANA conditions are common in HIV-related illness, in general, there are limited data to determine if the comorbidities (especially neuropsychiatric) are the result of the virus acting in the CNS, magnified by cART treatment, or comorbid with other chronic, inflammatory illness. The evidence favors an “accelerated aging” phenotype (i.e., organ- and disease-specific), but the question of whether the HANA conditions are accelerated or accentuated has not been answered [45, 68]. Thus, more research is needed addressing these possibilities to determine appropriate intervention targets.

Behavioral interventions that target comorbidities in the context of HIV have been limited to only one research group, employing tele-therapy or coping improvement and interpersonal support randomized controlled trial to relieve HIV-associated depressive symptomatology [88–90]. This group has further analyzed and characterized tele-therapy treatment efficacy for older HIV-infected persons in several ways: moderating roles of gender [91], sexual identity [92], and baseline depressive symptoms [93]; longitudinal sexual behavior patterns [94]; and trajectories of depression symptom relief [95]. This type of moderator (and mediational) analytic research of phenotypically defined aging/HIVgroups suggests possible improvements to interventions through further study of participant characteristics and/or intervention components and conditions for successful intervention trials. However, the preceding studies await replication by others to address generalizability and ecological validity, paving the way to developing novel intervention targets. The efficacy of these psychosocial interventions might be strengthened by combining with biological interventions since chronic inflammation (through a variety of mechanisms) contributes to HANA comorbidities in the elderly [82, 96].

### Cognitive Aging/HIV

HAND confers an increased risk for early mortality, independent of medical predictors [97]. As people age with HIV infection, the synergistic effects of aging and HIV may translate into more vulnerability to developing cognitive deficits that impact everyday activities over time [83]. A common intervention strategy has been to develop interventions that target specific cognitive deficits involved in daily functional activities [98]. HIV cognitive aging intervention targets are also suggested by neurocognitive specificity, e.g., interaction effects between HIV infection and age on motor speed, orientation, registration, and recall [99]. Some neuroimaging studies also identified the interaction effects between HIV infection and aging in selective brain functions [100, 101].

Because cognitive functions begin to decline in middle age or even earlier [102], it will be important for future studies to initiate interventions during middle age, rather than rely upon interventions in—later age when the negative cognitive consequences of dementing pathologic changes are generally detectable. This is particularly important in HIV infection, where “older age” has been set by the CDC as 50 years of age or older and the majority of patients fall in the 50–59-year-old age range. Such declines may take a long time to present clinically, but it is important to recognize that sub-clinical changes like metabolic alterations, chronic inflammation, and oxidative stress may also lead to behavioral changes, which can include reduced physical activity, decreased nutritional status, and greater social isolation. Therefore, interventions in mid-life (or earlier) may help prevent cognitive problems in aging with HIV infection [103].

HIV behavioral interventions for cognitive aging have been limited and mostly focused on cognitive rehabilitation of the speed of information processing [104, 105]. The studies show proof of concept for this strategy, and although the intervention covered a short time span, findings are encouraging because of the specificity of this intervention for informational processing speed abilities (no benefits for executive function). Cognitive improvement by enhancing or re-establishing information processing speed represents a restorative approach that relies upon neuroplasticity to encourage more effective neural organization. Cognitive decline may be addressed by developing interventions aimed at improving cognitive reserve [104, 105]. Engaging in stimulating cognitive activities (reading, social engagement, employment) can promote neuroplasticity, which, over time, encourages better cognitive reserve and also facilitates better neurocognitive functioning in general. When cognitive function cannot be restored, alternative compensatory approaches may be useful which involve the utilization of external methods (e.g., mnemonics or spaced-retrieval) or means to work around a deficit with the goal of improving cognitive functioning by supporting intact cognitive processes. This might be particularly effective for HIV-associated cognitive aging deficits in view of evidence that compensatory methods are generally more easily generalized to daily activities than restorative ones [106]. Cognitive rehabilitation techniques, whether restorative or compensatory, are not based alone on issues related to cognitive decline but are also mediated by engagement in treatment, and what can be done to enhance patient adherence for cognitive rehabilitation therapies to obtain greater cognitive and functional benefits from the treatment itself.

## Summary and Directions

We reviewed evidence for the main premise that chronological age alone is insufficient to represent aging effects in HIV research across the spectrum of aging. The phenotype concept was featured as a way to differentiate among relevant subsets of older adults with HIV infection.

The following conclusions emerge from this review.

1. *The comorbid and cognitive aging phenotypes are distinct, yet overlapping*. A distinct profile has been confirmed by cluster analyses and other statistical differentiation methods. However, the commonalities and interactions are many. This is similar to the conceptual debate about frailty as a single or multiple phenotype with different clusters of risk factors and etiologies [107]. The numerous non-AIDS comorbidities that contribute to cognitive status have posed a dilemma for clinicians and scientists to delineate the role of the virus from other influences [19].
2. *Associative relationships are the rule for HIV relationships between comorbid and cognitive aging and for comorbid or cognitive aging with other aging phenotypes.* While cognitive aging is frequently associated with comorbid aging in HIV infection, an aging-specific cognitive phenotype that is distinguishable from a variety of comorbid conditions has been reported in non-HIV/AIDS geriatric volunteers [108]. Therefore, comorbid and cognitive aging in HIV infection may not always co-exist but can be found individually and in various combinations. Because of the multiplicity of relevant variables, research should employ statistical procedures like structural equation modeling to separate out the contribution of individual variables. More studies of temporal relationships among phenotypes are needed to provide an additional layer of meaning in identifying bidirectionality and protection for the interpretation of sequential relationships.
3. *HIV behavioral interventions for both comorbid aging and cognitive aging have been limited.* Group tele-therapy focused on coping skills enhancement has been beneficial for comorbid aging and remediation training for cognitive aging. For these phenotypes, research is needed on other intervention modalities and should be expanded by different research groups. Broad replication is required for these studies to be considered representative and applicable to other settings. The mechanism by which these phenotypic-based effects may be exerted on older HIV-infected populations is also uncertain, although inflammatory mechanisms through a variety of processes are probably involved for comorbid and cognitive aging and cognitive reserve processes for cognitive aging. An important gap in phenotype-targeted interventions is research along each step of the HIV care continuum (i.e., testing/diagnosis, care linkage, engagement, treatment, viral suppression) to optimize the likelihood of viral suppression in older adults living with HIV/AIDS.
  Taken together, the reviewed findings on HIV/aging phenotypes suggest at least three paths for research progress: *clinical and behavioral specification, biological mechanisms, and intervention targets*.
  The approach, *clinical and behavioral specification*, has the goal of obtaining more precise definitions and improving specificity within the aging population to spawn advances in diagnosis, pathophysiology, and prevention/treatment, as well as to improve our understanding of the aging process. Evidence for using distinct phenotypic clusters for improved clinical and diagnostic assessment is suggested by diversity not only between phenotypes but also within a given phenotype (e.g., clusters of cognitive phenotypic subsets in older adults). This should provide greater precision in understanding each patient’s clinical features (e.g., comorbidities) and for selecting the most salubrious treatment for each individual patient. Thus, the pursuit of the long-term goal of increased homogeneity and specificity in HIV infection with aging is analogous to the Research Domain Criteria initiative to improve neurobiological validity, clinical prediction, and treatment matching [109]. A corollary of this behavioral/neurobiological dimension approach is that research incorporating relevant functional outcome measures has been guided by the notion that underlying processes in comorbid conditions involve multiple organ systems and result from a complex and multifactorial causes that requires increased dialog between different disciplines.
  Given the overlap of clinical domains within given phenotypes (e.g., cognitive aging), a key research area (i.e., *biological mechanisms*) is potential connections between the clinical realm of declines/dysfunction and the hypothesized biological mechanisms that may underlie these clinical changes. The role of chronic inflammation in HIV infection, aging, and AIDS comorbidities is one of the most important biological areas of research. If it could be further demonstrated that specific aspects of chronic inflammation or other biological factors are predictors of AIDS comorbidities in the elderly, then this would open additional avenues of prevention research to target these predictors (including host genetic predictors) before HIV/AIDS comorbidities emerge. The biological approach may yield genetically more homogeneous endophenotypes based on using different parent phenotypes to define and stratify older HIV-infected adults into clinical sub-groups. Evidence for this approach might be obtained by more narrowly defining the phenotype to identify more genetically similar sub-groups which may in turn increase power to detect biomarkers and genetic factors influencing risk.
  The *targeted integrated* approach simultaneously targets the multiple phenotypes that are associated with one another through a multi-level integrated approach. This is not only more practical and economical than targeting one phenotype at a time but also is consistent with contemporary high-impact prevention such as combination prevention strategies. Both approaches involve similar methodological challenges—delivering a coordinated package of interventions that matches the epidemiologic profile of a target population; delivering that package at the population level; and evaluating safety, acceptability, coverage, and effectiveness [110]. Within an integrated approach, it remains necessary to differentiate among the complexity of health care needs for patients with different phenotypes (e.g., comorbidity may or may not be present in frail or disabled aging individuals) to enable coordination of care among multiple providers. While the integrated approach may be preferred for interacting phenotypes, such as comorbid with cognitive aging and frailty with disability, a single domain intervention approach may be appropriate for those phenotypes that may exist, by and large, independently (e.g., successful aging) or for analyzing the impact of new interventions/treatments. However, addressing multiple phenotypic targets may potentially dilute the integrated intervention effects on any single outcome, especially when the problem behaviors do not share common influencing factors that the interventions were intended to address. Outcomes of multi-component interventions (bundled) are sometimes challenging to interpret because of difficulty in identifying the effective component(s), whereas factorial designs can efficiently reveal interactions among interventions, although such trials are often difficult to implement. As new discoveries and pathways are identified using complex systems analysis, functional genomics, and other emerging techniques, rigorous clinical trials will be necessary to demonstrate benefit of specific interventions. Comparative effectiveness research, using large HIV-related databases and emerging samples of older adults, may also offer another way for studying which treatments/interventions work best, for whom, and in what circumstances. Furthermore, with the exponential increase in quantity and complexity of the data available, it will be increasingly important to develop tools that enable clinicians to use this new information in a manner that will be reproducible, lead to more credible clinical research than currently possible, and enable translation of research into practice.
  With the above discussion of the three paths for research progress, we suggest some questions within each research path (Table 3).
  In conclusion, we re-emphasize that the multi-determined nature of the aging process provides a framework for physiological organization by which aging is mediated by multi-system dysfunction and the associated manifestation of heterogeneous, yet inter-related and interacting, behavioral phenotypes. The notion of interacting behavioral phenotypes has been similarly proposed for other aspects of the aging process, such as the integration of aging-related biomarkers into regulatory networks [111] and of interaction networks to identify aging-related genes, proteins, and pathways [112]. However, the phenotypic approach has not previously been given its due attention regarding age-dependent effects in the aging literature. Despite its empirical promise and utility in HIV and aging research, a cautionary note is in order. A schema of separate, singular phenotypes may accurately describe only a portion of the HIV and aging population, and there appears to be heterogeneity within and across groups typically considered homogeneous. We need to evaluate whether there are separate phenotypes with multiple risk factors, a common ultimate cause, both of these, or multiple phenotypes for a separate profile (e.g., frailty) with different constellations of risk factors, etiologies, and natural histories. Future research needs to build on the evolving ability to distinguish and untangle interacting phenotypes (e.g., cognition from comorbidity, disability from frailty), to refine their definitions and criteria, to develop standardized approaches to screening, and risk assessment, to identify physiological or molecular systems that are dysregulated, and to gain knowledge of interventions that prevent onset of and reverse adverse HIV-related outcomes.

**Table 3:** Suggested research questions for future research on HIV-related aging phenotypes

Approach	Research aims/questions
*Clinical and behavioral specification*	1. Identify differentiating characteristics and determinants of HIV/aging phenotypes;
	2. Examine impact of specific components for a given HIV-related aging phenotype on dysfunctions;
	3. Identify distinctive phenotypic clusters for improved clinical and diagnostic assessment between and within HIV-related aging phenotypes and facilitate development of targeted remediation and compensatory interventions;
	4. Predict prognosis and treatment outcomes for HIV-related aging phenotypes
	5. Assess and measure function for HIV-related aging phenotypes and validate as predictors of relevant outcomes in HIV-infected populations.
*Biological mechanisms*	1. Identify clusters of risk factors for specific HIV-related aging phenotypes as potential targets for preventive or therapeutic strategies.
	2. Develop reliable biomarkers of HIV-related aging phenotypes and link biomarkers to clinical events including response to anti-inflammatory treatments and associated morphologic improvements.
	3. Determine specific mechanism underlying chronic inflammation as predictors of HIV-related comorbid aging to potentially target these predictors (e.g., host genetic predictors) before AIDS comorbidities emerge.
	4. Identify physiological vulnerabilities central to HIV-related aging phenotypes including poor response to stressors and mechanisms that underlie subclinical components.
	5. Employ computational biology strategies (e.g., nonlinear methods) to address multiple interrelated physiological and molecular systems with clinical outcomes.
*Targeted intervention*	1. Improve understanding of epidemiologic contexts for HIV-related aging phenotype targeted interventions including accurate rates of testing, linkage, initiation and viral suppression that indicates gaps and targets for intervention;
	2. Enhance intervention research on multiple prevention messages fopr HIV-related aging phenotypes and expand with multilevel intervention strategies including individual, group, community, structural, and policy level strategies.
	3. Analyze most effective components of integrated interventions (and which integrated interventions work best, for whom, and in what circumstances) within overlapping interacting HIV-related aging phenotypes and develop most promising, cost-effective integrated interventions. (using pathway modeling or other dynamic systems approaches);
	4. Conduct predictor and moderator analyses to suggest ways in which older patients may be selected for different HIV interventions and suggest potential avenues for further development of the interventions for increasing their effectiveness within certain subgroups, identified by HIV-related aging phenotypes;
	5. Examine each step of HIV care continuum in older adults to optimize the likelihood of viral suppression within certain subgroups, identified by individual or interacting HIV-related aging phenotypes.
